# Patient-important outcomes in clinical trials of atopic diseases and asthma in the last decade: A systematic review^☆^

**DOI:** 10.1016/j.waojou.2023.100769

**Published:** 2023-04-30

**Authors:** Sandra Nora González-Díaz, Mariano García-Campa, Andrés Noyola-Pérez, Rosa-Ivett Guzmán-Avilán, Cindy Elizabeth de Lira-Quezada, Neri Álvarez-Villalobos, René Rodríguez-Gutiérrez, Carlos Macouzet-Sánchez

**Affiliations:** a Universidad Autónoma de Nuevo León, Centro Regional de Alergia e Inmunología Clínica, Hospital Universitario “Dr. José Eleuterio González”, Francisco I. Madero Avenue, Mitras Centro, ZC 64460, Monterrey, Mexico; b Plataforma Universidad Autónoma de Nuevo León, INVEST UANL-KER Unit Mayo Clinic, School of Medicine and University Hospital “Dr José E González”, Monterrey 64460, Mexico

**Keywords:** Allergy, Asthma, Patient-important outcomes, Allergic rhinitis

## Abstract

**Background:**

Randomized Clinical Trials (RCTs) are important tools to establish the effects of a given intervention. Investigators should focus on outcomes that patients perceive: patient-important outcomes (PIOs), clinical endpoints that patients value directly and reflect how they feel, function, or survive. However, it is easier to consider surrogated outcomes to reduce costs and achieve better-looking results. The problem with these outcomes is that they indirectly measure PIOs, which might not correlate linearly or translate reliably into a positive PIO.

**Methods:**

We systematically searched MEDLINE for atopic disease RCTs rated among the top 10 allergic diseases and general internal medicine journals from the last 10 years. Two independent reviewers worked in duplicate and independently to collect data from all eligible articles. We gathered information regarding the type of study, title, author information, journal, intervention type, atopic disease, and primary and secondary outcomes. We assessed the outcomes investigators used in RCTs of atopic diseases and asthma.

**Results:**

This quantitative analysis included n = 135 randomized clinical trials. The most studied atopic disease during the period selected was asthma (n = 69), followed by allergic rhinitis (n = 51). When divided by atopic disease, primary outcomes in RCTs valuing allergic rhinitis had the most significant proportion of PIOs (76.7), asthma surrogated outcomes (38), and asthma/allergic rhinitis laboratory outcomes (42.9). PIOs in allergic rhinitis trials had the most significant proportion of PIOs favoring the intervention (81.4), asthma had the greatest proportion of surrogated outcomes (33.3), and asthma/allergic rhinitis laboratory outcomes (40). When divided by atopic disease, trials studying atopic dermatitis and urticaria had the same proportion of PIOs (64.7) as their secondary outcomes. Asthma had the highest (37.5) surrogate outcomes. Journals of general/internal medicine had a greater proportion of PIOs, and a post hoc analysis showed a significant difference in the proportion and secondary outcomes that favored the intervention between PIOs and laboratory outcomes.

**Conclusion:**

Approximately 7.5 out of 10 primary outcomes in RCTs published in general/internal medicine are PIOs compared to 5 out of 10 primary outcomes in atopic disease journals. Investigators should focus on selecting patient-important outcomes in their clinical trials to establish clinical guidelines with better-quality recommendations that impact patients’ life and values.

**Registration:**

International Prospective Register of Systematic Reviews (PROSPERO, NIHR) ID: CRD42021259256.

## Introduction

**Fig. 1:**
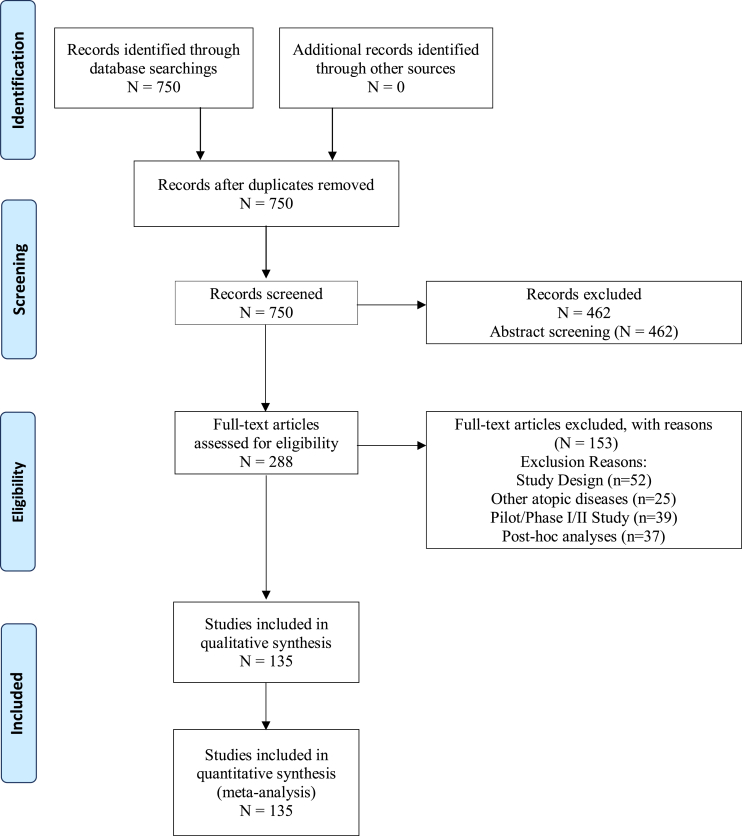
Prisma with details

Medical research, especially clinical trials, always tries to improve patients’ conditions and delve further into unknown diseases. Because of the development of the enzyme-linked immunosorbent assay (ELISA) and, more recently, the polymerase chain reaction (PCR), investigators could be tempted to describe whole chemical metabolic pathways, biochemical parameters, or even cytokines in allergic or immunologic diseases. Clinical trials valuing interventions that address symptoms or health conditions should be designed around the idea that: 1) these interventions are reliable, replicable, and accessible for most of the population, and 2) they focus on communicating and solving patients’ most essential needs. Likewise, considering the implications of conducting a randomized clinical trial (RCT) and being one of the most important tools to establish the effects of a given intervention, investigators should focus on the outcomes patients perceive as necessary: patient-important outcomes (PIOs). PIOs have been defined as characteristics or variables that represent how patients feel, function, or survive, in contrast to surrogated outcomes, which try to parallel PIOs.^1^ The need to use PIOs lies fundamentally in high-quality evidence that is important for patients to aid clinicians in decision-making. These outcomes have been explored in other medical conditions, such as diabetes, critically ill adults, and lung transplantation.^2^, ^3^, ^4^ In RCTs of diabetes, authors reported that only 18% of primary outcomes were considered PIOs. Similarly, in RCTs of critically ill adults, only 24% were PIOs, and in lung transplantation, 23.5%. This finding shows a clear trend of RCTs of different conditions that do not prioritize PIOs. There are some disadvantages to incorporating PIOs in clinical trials, such as higher costs. It is easier to use surrogate outcomes to reduce costs and have better-looking results. The problem with these outcomes is that they indirectly measure PIOs, and these measures might not correlate linearly or reliably translate into a positive PIO.^5^ The proportion of patient-important outcomes in atopic diseases and asthma RCTs, to our knowledge, is unknown. Therefore, we performed a systematic review to assess the outcomes investigators use in RCTs of atopic diseases (see Fig. 1).

## Methods

### Study design review

This protocol adheres to the Preferred Reporting Items for Systematic Review and Meta-Analysis (PRISMA) statement and has successfully been registered in the International Prospective Register of Systematic Reviews (PROSPERO, NIHR) under the ID: CRD42021259256.^6^

### Eligibility criteria

We included randomized clinical trials (RCTs) from June 2011 to June 2021 published in the following journals: the New England Journal of Medicine (NEJM), the Journal of the American Medical Association (JAMA), the British Medical Journal (BMJ), Annals of Internal Medicine, JAMA Internal Medicine, the Journal of Allergy and Clinical Immunology (JACI), Allergy, JACI: In-Practice, Clinical and Translational Allergy (CTA), and Annals of Allergy Asthma & Immunology. Selected RCTs must have defined and reported primary and secondary outcomes and pediatric or adult population with any of the following atopic diseases: asthma, allergic rhinitis, atopic dermatitis (eczema), and urticaria. Only RCTs in English or Spanish were considered. If studies included multiple outcomes as their primary or secondary outcomes, all were included. Composite outcomes were assessed in the same manner as a multiple-component primary outcome if the authors broke down the effect of each outcome.

Exclusion criteria were phase I/II clinical trials, pilot RCTs, and studies that did not measure the individual effect of the components of their composite outcome. If outcomes were measured in patients and *in vitro* samples, only outcomes in an *in vivo* clinical scenario were considered. We defined patient-important outcomes as any event or outcome directly related to improving prevention, quality of life (QoL), functional status, symptoms, or overall health and reducing mortality, including symptom scales, QoL, adverse events, disease-related exacerbations, missed work/school days, and hospitalizations. The outcome measurement should be validated; if not validated, it was considered a surrogate outcome. Surrogate outcomes were considered as any outcome that indirectly measured a patient-important outcome. Laboratory outcomes were defined as parameters that do not indirectly measure patient-important outcomes.

### Search strategy & data management

An experienced librarian helped us design and conduct the search strategy. We searched Medline from June 2011 through June 2021 and complemented this initial strategy by screening the online journal websites for potentially missed RCTs. Additionally, 2 authors, independently and in duplicate, searched manually for RCTs in each journal. All search results were uploaded to EndNote X8 for de-duplication. The resulting studies were uploaded to Distiller Systematic Review (DSR) software for title/abstract and full-text screening.

### Study selection process

Study selection took place in 2 phases (title/abstract and full-text screening). Two reviewers worked independently and in duplicate to assess the eligibility of the studies in each review phase. Chance-adjusted inter-rater agreement was assessed at each phase using the Kappa statistic. Before each screening phase, a pilot test with a random sample of studies from the search strategy results was carried out to standardize the reviewers’ criteria. Disagreements were discussed, and criteria were adapted if necessary. The pilot test was repeated until a Kappa index >0.70 was reached.^7^ In the first phase, the title and abstract of all the studies from the search strategy were screened. Reviewers selected the eligible articles based on the inclusion criteria. Discordant decisions passed to the full-text phase to achieve a highly sensitive selection. Eligibility was then assessed with the full-text screening. Disagreements between the reviewers during this second phase were resolved by consensus, and if not achieved, a third reviewer arbitrated. We documented the number of included and excluded articles and the reasons for the exclusion of each study during the entire process.

## Data collection

Two independent reviewers worked in duplicate and independently to collect data from all eligible articles using a web-based data extraction form. We gathered information regarding the type of study, title, author information, journal, type of intervention, type of atopic disease, and primary & secondary outcome. Disagreements were resolved by consensus; if an agreement was not reached, a third reviewer made the final decision. Before this process, the 2 reviewers conducted a pilot test independently and in duplicate. Disagreements were resolved by consensus; if an agreement was not reached, a third reviewer made the final decision. The reviewers gave feedback about any suggested adjustments, and if necessary, they were applied to the preliminary extraction form.

### Missing data

If data necessary for our outcomes were missing or unclear, the corresponding author was contacted via e-mail to clarify. Then, after 10 days, a second e-mail was sent to non-responders. If there was still no response, other authors were contacted. Finally, if no response was received after all attempts, the study was excluded. Every contact was documented.

### Data synthesis

The included studies were described as a narrative synthesis in a table including the author's information, type of intervention, type of atopic disease targeted, primary and secondary outcomes, and if the primary/secondary outcome favors the intervention. We used SPSS version 25 for statistical analysis.^8^

The outcomes were summarized in proportions according to the classification of the primary and secondary outcomes. If possible, a Chi-square test was performed to evaluate the difference between the proportions of patient-important, surrogate, and laboratory outcomes in the included RCTs’ primary and secondary outcomes. A post-hoc analysis was performed based on the two groups with the highest standardized residuals. Finally, a Bonferroni correction to the p-value was performed to assess statistical differences.^9^ For the rest of the statistical tests, a p ≤ 0.05 was a significant difference.

## Results

A total of 135 randomized clinical trials were included in the quantitative analysis; 112 were selected from the top 5 atopic disease journals. The majority (n = 63) were funded by for-profit organizations, followed by governmental (n = 24) and mixed funding (n = 22) by either for-profit, non-profit, or governmental organizations. Only 5 and 1 were RCTs suspected of conflict of interest from for-profit and not-for-profit organization(s), respectively.

The most studied atopic disease during the selected period was asthma (n = 69), followed by allergic rhinitis (n = 51). RCTs with a parallel design (n = 116) had drug against an active drug (n = 22), drug vs. placebo (n = 40), educational/lifestyle intervention (n = 15), immunotherapy vs. placebo (n = 23), acupuncture vs. sham acupuncture (n = 2), dietary intervention (n = 2), device intervention (n = 2), and other interventions (n = 9). Similarly, RCTs with a cross-over design (n = 19) had interventions, such as drug vs. placebo (n = 12), educational/lifestyle (n = 2), and others (n = 5). Primary outcomes were identified in all included RCTs (n = 246): patient-important outcomes (n = 141), surrogate outcomes (n = 62), and laboratory outcomes (n = 43); of these, PIOs (n = 110), and surrogate (n = 39) and laboratory outcomes (n = 23) favored the intervention defined by the authors. In contrast, secondary outcomes were identified in 863: PIOs (n = 437), surrogate (n = 229), and laboratory outcomes (n = 197), where PIOS (n = 261), surrogate (n = 111), and laboratory (n = 76) outcomes favored the intervention.

When divided by atopic disease (Table 1), primary outcomes in RCTs valuing allergic rhinitis had the most significant proportion of PIOs (76.7%), asthma of surrogated outcomes (38%), and asthma/allergic rhinitis laboratory outcomes (42.9%). A post hoc analysis showed that the different proportions between surrogated and laboratory outcomes from RCTs studying asthma and asthma/allergic rhinitis were not statistically significant. Similarly, in allergic rhinitis trials, PIOs had the most significant proportion favoring the intervention (81.4%). Asthma had the greatest proportion of surrogate (33.3%) and asthma/allergic rhinitis in laboratory outcomes (40%). Post hoc analysis showed no significant differences in surrogate and laboratory outcome proportions from asthma vs. asthma/allergic rhinitis. When divided by type of intervention, dietary interventions valued only PIOs, drug vs placebo had the most significant proportion of surrogated outcomes (38.3%), and acupuncture vs. sham acupuncture, laboratory outcomes (57.1%). Post hoc analysis showed no significant difference between the proportions of surrogated and laboratory outcomes from acupuncture vs. sham acupuncture and drug vs placebo interventions. No differences in the proportion of primary outcomes favored the intervention when stratified by type of intervention. When divided by type of funding, RCTs funded by the government had the most significant proportion of PIOs (65.9%), studies with no funding recorded the highest proportion of surrogated outcomes (28.5%), and mixed funding, laboratory outcomes (32.1%). Post hoc analysis showed no significant difference in surrogate and laboratory outcomes between RCTs with no funding vs. mixed funding. RCTs published in the top 5 selected general/internal medicine journals had more significant proportions of PIOs than selected allergy journals (76.5% vs. 54.2%). Post hoc analysis showed no difference between PIOs and laboratory outcomes. Similarly, no differences were observed in outcomes favoring the intervention. In RCTs with a parallel design, compared to cross-over, post hoc analysis showed a significant difference between PIOs and laboratory outcomes and in the proportion of PIOs favoring the intervention compared to laboratory outcomes.

**Table 1:** Description of 135 randomized clinical trials primary Outcomes'

Trial Characteristics										
	Primary Outcome					Primary Outcome Favored the Intervention				
	PIO	Surrogated	Laboratory	p-value	Post-hoc	PIO	Surrogated	Laboratory	p-value	Post-hoc
**Atopic Diseases**										
Asthma	50 (50)	**38 (38)**	**12 (12)**	**<0.0001**	0.02	34 (49.3)	**23 (33.3)**	**12 (17.4)**	**<0.0001**	0.052
Allergic Rhinitis	69 (76.7)	17 (18.9)	4 (4.4)			57 (81.4)	13 (18.6)	0		
Atopic Dermatitis	6 (50)	2 (16.7)	4 (33.3)			6 (60)	0	4 (40)		
Urticaria	7 (63.6)	2 (18.2)	2 (18.2)			6 (75)	1 (12.5)	1 (12.5)		
Asthma/Allergic Rhinitis	9 (42.9)	**3 (14.3)**	**9 (42.9)**			7 (46.7)	**2 (13.3)**	**6 (40)**		
**Trial Design**										
Parallel	**132 (64.7)**	46 (22.5)	**26 (12.7)**	**<0.0001**	**<0.00001**	**103 (67.3)**	32 (20.9)	**18 (11.8)**	**0.03**	0.34
Cross-over	**9 (21.4)**	16 (38.1)	**17 (40.5)**			**7 (36.8)**	7 (36.8)	**5 (26.3)**		
**Type of intervention**										
Acupunture vs Sham Acupunture	2 (28.6)	**1 (14.3)**	**4 (57.1)**	**<0.0001**	0.009	2 (66.7)	1 (33.3)	0	0.051	
Dietary Intervention	2 (100)	0	0			1 (100)	0	0		
Drug vs Active Drug	24 (80)	6 (20)	0			18 (81.8)	4 (18.2)	0		
Drugs vs Placebo	37 (54.4)	**26 (38.2)**	**5 (7.4)**			30 (62.5)	15 (31.3)	3 (6.3)		
Education/Lifestyle Intervention	21 (46.7)	22 (24.4)	13 (28.9)			16 (66.7)	5 (20.8)	3 (12.5)		
Immunotherapy vs Placebo	42 (66.7)	8 (12.7)	13 (20.6)			32 (68.1)	5 (10.6)	10 (21.3)		
Other	13 (44.8)	8 (27.6)	8 (27.6)			11 (40.7)	9 (33.3)	7 (25.9)		
**Funding**										
No Funding	18 (46.2)	**15 (38.5)**	**6 (15.4)**	**0.001**	0.019	17 (54.8)	8 (25.8)	6 (19.4)	0.149	
For-No-Profit Organization(s)	19 (52.8)	8 (22.2)	9 (25)			14 (58.3)	4 (16.7)	6 (25)		
For-Profit Organization(s)	50 (64.9)	24 (31.2)	3 (3.9)			47 (70.1)	18 (26.9)	2 (3)		
Goverment	27 (65.9)	6 (14.6)	8 (19.5)			16 (66.7)	4 (16.7)	4 (16.7)		
Mixed	27 (50.9)	**9 (17)**	**17 (32.1)**			16 (61.5)	5 (19.2)	5 (19.2)		
**Journal**										
Top 5 Selected General/IM	**26 (76.5)**	7 (20.6)	**1 (2.9)**	**0.022**	0.006	21 (75)	6 (21.4)	1 (3.6)	0.602	
Top 5 Selected Allergy	**115 (54.2)**	55 (25.9)	**42 (19.8)**			89 (66.9)	33 (24.8)	11 (8.3)		

When divided by atopic disease (Table 2), trials studying atopic dermatitis and urticaria had the same proportion of PIOs (64.7%) as their secondary outcomes, and asthma had the highest (37.5%) in surrogate outcomes. Post hoc analysis showed a statistically significant difference between surrogate and laboratory outcomes in asthma vs. asthma/allergic rhinitis. In contrast, allergic rhinitis had the greatest proportion of PIOs favoring the intervention. Post hoc analysis showed a significant difference in surrogate and laboratory outcomes in asthma and asthma/allergic rhinitis trials. When split by type of intervention, drug vs active drug had the greatest proportion of PIOs (66.7%), and post-hoc analysis showed a significant difference between PIOs and laboratory outcomes in dietary interventions against drug vs. drug. Acupuncture vs sham acupuncture had the highest proportion of PIOs (100%) favoring the intervention. Government-funded studies had the highest PIOs proportion (60.9%). PIOs favoring the intervention (78.6%) and post hoc analysis showed a significant difference between PIOs and laboratory outcomes in studies with no funding vs. those funded by the government. Journals of general/internal medicine had a greater proportion of PIOs, and post hoc analysis showed a significant difference in the proportion and secondary outcomes that favored the intervention between PIOs and laboratory outcomes.

**Table 2:** Description of 135 randomized clinical trials secondary Outcomes'

Trial Characteristics										
	Secondary Outcome					Secondary Outcome Favored the Intervention				
	PIO	Surrogated	Laboratory	p-vale	Post-hoc	PIO	Surrogated	Laboratory	p-value	Post-hoc
**Atopic Diseases**										
Asthma	183 (47.7)	**144 (37.5)**	**57 (14.8)**	**<0.0001**	**<0.00001**	88 (49.4)	**68 (38.2)**	**22 (12.4)**	**<0.0001**	**<0.00001**
Allergic Rhinitis	185 (52.4)	63 (17.8)	105 (29.7)			142 (66.4)	33 (15.4)	39 (18.2)		
Atopic Dermatitis	33 (64.7)	6 (11.8)	12 (23.5)			14 (70)	3 (15)	3 (15)		
Urticaria	33 (64.7)	15 (30)	2 (4)			17 (68)	7 (28)	1 (4)		
Asthma/Allergic Rhinitis	3 (15.8)	**1 (5.3)**	**15 (78.9)**			0	**0**	**11 (100)**		
**Trial Design**										
Parallel	**413 (52.7)**	205 (26.1)	**166 (21.2)**	**<0.0001**	**0.00005**	**243 (60.6)**	98 (24.4)	**60 (15)**	**0.002**	0.001
Cross-over	**24 (30.4)**	24 (30.4)	**31 (39.2)**			**18 (38.3)**	13 (27.7)	**16 (34)**		
**Type of intervention**										
Acupunture vs Sham Acupunture	10 (90.9)	1 (9.1)	0	**<0.0001**	**<0.00001**	8 (100)	0	0	**<0.0001**	0.095
Dietary Intervention	**2 (8.7)**	2 (8.7)	**19 (82.6)**			0	6 (75)	2 (25)		
Drug vs Active Drug	**96 (66.7)**	43 (29.9)	**5 (3.5)**			38 (70.4)	15 (27.8)	1 (1.9)		
Drugs vs Placebo	205 (57.4)	90 (25.2)	62 (17.4)			145 (67.8)	45 (21)	24 (11.2)		
Education/Lifestyle Intervention	43 (53.8)	33 (41.3)	4 (5)			19 (55.9)	14 (41.2)	1 (2.9)		
Immunotherapy vs Placebo	56 (37.8)	34 (23)	58 (39.2)			**37 (45.7)**	18 (22.2)	**26 (32.1)**		
Other	25 (25.5)	24 (24.5)	49 (50)			**14 (29.2)**	12 (25)	**22 (45.8)**		
**Funding**										
No Funding	**25 (32.5)**	19 (24.7)	**33 (42.9)**	**<0.0001**	**<0.00001**	15 (41.7)	13 (36.1)	8 (22.2)	**0.005**	0.007
For-No-Profit Organization(s)	241 (53.3)	103 (22.8)	108 (23.9)			10 (71.4)	4 (28.6)	0		
For-Profit Organization(s)	20 (41.7)	29 (39.6)	9 (18.8)			171 (59.2)	65 (22.5)	53 (18.3)		
Goverment	**81 (60.9)**	34 (25.6)	**18 (13.5)**			**33 (78.6)**	**6 (14.3)**	3 (7.1)		
Mixed	70 (45.8)	54 (35.3)	29 (19)			**32 (47.8)**	**23 (34.3)**	12 (17.9)		
**Journal**										
Top 5 Selected General/IM	**99 (64.7)**	49 (32)	**5 (3.3)**	**<0.0001**	**<0.00001**	**52 (67.5)**	23 (29.9)	**2 (2.6)**	**0.001**	**0.00007**
Top 5 Selected Allergy	**338 (47.6)**	180 (25.4)	**192 (27)**			**209 (56.3)**	88 (23.7)	**74 (19.9)**		

## Discussion

Our results show that 7.5 out of 10 primary outcomes in RCTs published in general/internal medicine are PIOs compared to 5 out of 10 primary outcomes in atopic disease journals. This finding could be due to advanced techniques in immunology addressing diseases from a molecular level. Conversely, 6 out of 10 secondary outcomes in RCTs published in general/internal medicine are PIOs compared to 5 out of 10 in atopic disease journals. This information could be misleading due to the nature of secondary outcomes. Since most RCTs base their statistical power on primary outcomes, these outcomes can be of any type. Researchers and sponsors should design and fund clinical trials that value outcomes that favor the preferences and values of patients. Our study shows that studies with no funding had the lowest proportion of PIOs, evidencing a preference of organizations and governments to fund trials that value them but still with a balance in trying to improve science and the clinical field. Leaving aside surrogate or laboratory outcomes is not always possible, but focusing on including patient-important outcomes in those trials could also give clinical and patient contexts. With this, clinical guidelines would have better quality recommendations that directly impact the patient's life.

One of the possible solutions for RCTs to reflect PIOs is patient and public involvement (PPI) in almost every trial design phase. Including patients in the development of the recruitment process could help investigators select a true targeted population, especially when representing minorities.^10^^,^^11^ This reduces the possibility of selecting a specific population recruited in high-specialty tertiary-care centers. PPI is also important when selecting PIOs and the comparator in clinical trials to reduce the loss to follow-up by generating a patient-friendly care ecology. However, evidence on the reasons for leaving clinical trials is limited. A survey done in a French population from both public and private centers showed that only 48.3% of participants trusted pharmaceutical companies involved in research, and 35.5% would not participate in trials designed by the pharma-industry due to distrust in respiratory disease-related trials.^12^ PPI is crucial to improve clinical trials and generate effective communication between sponsors, investigators, and patients.

Some limitations of this systematic review are that we could attribute our results to the fact that general medicine journals try to target all levels of care (primary-tertiary) and, thus, consider PIOs rather than basic science or immunology seen in high-specialty journals of atopic diseases. On the other hand, it is difficult to isolate a population of allergic asthma and normally are included in a broader classification of asthma. We believe that including all asthma classifications is relevant for this study since clinicians need to have the differential diagnosis in mind.

## Conclusions

Approximately 7.5 out of 10 primary outcomes in RCTs published in general/internal medicine are PIOs compared to 5 out of 10 primary outcomes in atopic disease journals. Investigators should focus on selecting patient-important outcomes in their clinical trials to develop clinical guidelines with better quality recommendations that impact the patient's life and values.

## Funding

No funding was received for this project.

## Availability of data and materials

The data underlying this article will be shared upon reasonable request to the corresponding author.

## Author contributions

NAV and RRG designed and performed the search strategy. MGC and ANP extracted and analyzed the data. MGC, ANP, and CMS interpreted the data. All authors contributed to in the design of the protocol, writing and, approving the final version of this article. SNGD provided additional administrative support and guidance throughout the process.

## Ethics approval

No Ethics approval was required for this study.

## Authors’ consent for publication

All authors consent for the publication of the manuscript entitled “Patient-Important outcomes in clinical trials of atopic diseases in the last decade: A systematic review.” We read the final version and give our consent for the article to be published in the World Allergy Organization Journal.

## Declaration of competing interest

Authors declare no competing interest.
