# Supplementary material for: Patient-important outcomes in clinical trials of atopic diseases and asthma in the last decade: A systematic review
Source: World Allergy Organ J. 2023 Apr 30;16(4):100769. doi: 10.1016/j.waojou.2023.100769 (PMC10172603; doi:10.1016/j.waojou.2023.100769)
Supplement: Multimedia component 1 [file mmc1.pdf]

Supplemental Figure 1: Search Strategy

Search Number	Query	Search Details	Results
8	#5 AND #6	((((((((((randomized controlled trial[Publication Type]) OR (controlled clinical trial[Publication Type])) OR (randomized[Title/Abstract])) OR (placebo[Title/Abstract])) OR (clinical trials as topic[MeSH Terms])) OR (randomly[Title/Abstract])) OR (trial[Title])) NOT ((animals[MeSH Terms]) NOT (humans[MeSH Terms]))) AND (((((((((("N Engl J Med,"[Journal]) OR ("J Am Med Assoc."[Journal])) OR ("Br Med J"[Journal])) OR ("Ann Intern Med."[Journal])) OR ("JAMA Intern Med."[Journal])) OR ("J Allergy Clin Immunol Pract."[Journal])) OR ("Allergy."[Journal])) OR ("J Allergy Clin Immunol Pract."[Journal])) OR ("Clin Transl Allergy."[Journal])) OR ("Ann Allergy Asthma Immunol."[Journal]))) AND (("Asthma " or "Asthma, Bronchial" or "Asthmas" or "Bronchial Asthma " or "Rhinitis, Allergic" or "Allergic Rhinitides" or "Allergic Rhinitis" or "Rhinitides, Allergic" or "Dermatitis, Atopic" or "Atopic Dermatitides" or "Atopic Dermatitis" or "Atopic Eczema" or "Atopic Neurodermatitides" or "Atopic Neurodermatitis" or "Dermatitides, Atopic" or "Disseminated Neurodermatitides" or "Disseminated Neurodermatitis" or "Eczema, Atopic" or "Eczema, Infantile" or "Infantile Eczema" or "Neurodermatitides, Atopic" or "Neurodermatitides, Disseminated" or "Neurodermatitis, Atopic" or "Neurodermatitis, Disseminated" or "Urticaria " or "Hives" or "Urticarias"))) AND (y_10[Filter])	750

7	#5 AND #6	(((((((((randomized controlled trial[Publication Type]) OR (controlled clinical trial[Publication Type])) OR (randomized[Title/Abstract])) OR (placebo[Title/Abstract])) OR (clinical trials as topic[MeSH Terms])) OR (randomly[Title/Abstract])) OR (trial[Title])) NOT ((animals[MeSH Terms]) NOT (humans[MeSH Terms]))) AND ((((((((((("N Engl J Med,"[Journal]) OR ("J Am Med Assoc."[Journal])) OR ("Br Med J"[Journal])) OR ("Ann Intern Med."[Journal])) OR ("JAMA Intern Med."[Journal])) OR ("J Allergy Clin Immunol Pract."[Journal])) OR ("Allergy."[Journal])) OR ("J Allergy Clin Immunol Pract."[Journal])) OR ("Clin Transl Allergy."[Journal])) OR ("Ann Allergy Asthma Immunol."[Journal]))) AND (("Asthma " or "Asthma, Bronchial" or "Asthmas" or "Bronchial Asthma " or "Rhinitis, Allergic" or "Allergic Rhinitides" or "Allergic Rhinitis" or "Rhinitides, Allergic" or "Dermatitis, Atopic" or "Atopic Dermatitides" or "Atopic Dermatitis" or "Atopic Eczema" or "Atopic Neurodermatitides" or "Atopic Neurodermatitis" or "Dermatitides, Atopic" or "Disseminated Neurodermatitides" or "Disseminated Neurodermatitis" or "Eczema, Atopic" or "Eczema, Infantile" or "Infantile Eczema" or "Neurodermatitides, Atopic" or "Neurodermatitides, Disseminated" or "Neurodermatitis, Atopic" or "Neurodermatitis, Disseminated" or "Urticaria " or "Hives" or "Urticarias"))	2,415
6	("Asthma " or "Asthma, Bronchial" or "Asthmas" or "Bronchial Asthma " or "Rhinitis, Allergic" or "Allergic Rhinitides" or "Allergic	("Asthma " or "Asthma, Bronchial" or "Asthmas" or "Bronchial Asthma " or "Rhinitis, Allergic" or "Allergic Rhinitides" or "Allergic	256,608

	Rhinitis" or "Rhinitides, Allergic" or "Dermatitis, Atopic" or "Atopic Dermatitides" or "Atopic Dermatitis" or "Atopic Eczema" or "Atopic Neurodermatitides" or "Atopic Neurodermatitis" or "Dermatitides, Atopic" or "Disseminated Neurodermatitides" or "Disseminated Neurodermatitis" or "Eczema, Atopic" or "Eczema, Infantile" or "Infantile Eczema" or "Neurodermatitides, Atopic" or "Neurodermatitides, Disseminated" or "Neurodermatitis, Atopic" or "Neurodermatitis, Disseminated" or "Urticaria " or "Hives" or "Urticarias")	Rhinitis" or "Rhinitides, Allergic" or "Dermatitis, Atopic" or "Atopic Dermatitides" or "Atopic Dermatitis" or "Atopic Eczema" or "Atopic Neurodermatitides" or "Atopic Neurodermatitis" or "Dermatitides, Atopic" or "Disseminated Neurodermatitides" or "Disseminated Neurodermatitis" or "Eczema, Atopic" or "Eczema, Infantile" or "Infantile Eczema" or "Neurodermatitides, Atopic" or "Neurodermatitides, Disseminated" or "Neurodermatitis, Atopic" or "Neurodermatitis, Disseminated" or "Urticaria " or "Hives" or "Urticarias")	
5	#3 AND #4	(("randomized controlled trial"[Publication Type] OR "controlled clinical trial"[Publication Type] OR "randomized"[Title/Abstract] OR "placebo"[Title/Abstract] OR "clinical trials as topic"[MeSH Terms] OR "randomly"[Title/Abstract] OR "trial"[Title]) NOT ("animals"[MeSH Terms] NOT "humans"[MeSH Terms])) AND ("n engl j med"[Journal] OR "j am med assoc"[Journal] OR "Br Med J"[Journal] OR "ann intern med"[Journal] OR "jama intern med"[Journal] OR "j allergy clin immunol pract"[Journal] OR "allergy"[Journal] OR "j allergy clin immunol pract"[Journal] OR "clin transl allergy"[Journal] OR "ann allergy asthma immunol"[Journal])	13,341
4	(((((((((("N Engl J Med,"[Journal]) OR ("J Am Med Assoc."[Journal])) OR ("Br Med J"[Journal])) OR ("Ann Intern Med."[Journal])) OR ("JAMA Intern Med."[Journal])) OR ("J Allergy Clin Immunol Pract."[Journal])) OR ("Allergy."[Journal])) OR ("J Allergy Clin Immunol Pract."[Journal])) OR ("Clin Transl Allergy."[Journal])) OR	"n engl j med"[Journal] OR "j am med assoc"[Journal] OR "Br Med J"[Journal] OR "ann intern med"[Journal] OR "jama intern med"[Journal] OR "j allergy clin immunol pract"[Journal] OR "allergy"[Journal] OR "j allergy clin immunol pract"[Journal] OR "clin transl allergy"[Journal] OR "ann allergy asthma immunol"[Journal]	249,986

	("Ann Allergy Asthma Immunol."[Journal])		
3	#1 NOT #2	("randomized controlled trial"[Publication Type] OR "controlled clinical trial"[Publication Type] OR "randomized"[Title/Abstract] OR "placebo"[Title/Abstract] OR "clinical trials as topic"[MeSH Terms] OR "randomly"[Title/Abstract] OR "trial"[Title]) NOT ("animals"[MeSH Terms] NOT "humans"[MeSH Terms])	1,358,286
2	(animals[MeSH Terms]) NOT (humans[MeSH Terms])	"animals"[MeSH Terms] NOT "humans"[MeSH Terms]	4,858,855
1	((((((randomized controlled trial[Publication Type]) OR (controlled clinical trial[Publication Type])) OR (randomized[Title/Abstract])) OR (placebo[Title/Abstract])) OR (clinical trials as topic[MeSH Terms])) OR (randomly[Title/Abstract])) OR (trial[Title])	"randomized controlled trial"[Publication Type] OR "controlled clinical trial"[Publication Type] OR "randomized"[Title/Abstract] OR "placebo"[Title/Abstract] OR "clinical trials as topic"[MeSH Terms] OR "randomly"[Title/Abstract] OR "trial"[Title]	1,468,651

Supplemental Table 1. Reference List of Included Randomized Clinical Trials

**Atopic Disease Journals**
Ali, A. M., Selim, S., Abbassi, M. M., & Sabry, N. A. (2017). Effect of alfacalcidol on the pulmonary function of adult asthmatic patients: A randomized trial. *Annals of allergy, asthma & immunology : official publication of the American College of Allergy, Asthma, & Immunology*, *118*(5), 557–563. https://doi.org/10.1016/j.anai.2017.02.014
Amakye, D. O., Davis, B. E., Martin, A. L., Peters, G. E., & Cockcroft, D. W. (2013). Refractoriness to inhaled mannitol 3 hours after allergen challenge. *Annals of allergy, asthma & immunology : official publication of the American College of Allergy, Asthma, & Immunology*, *111*(3), 182–184. https://doi.org/10.1016/j.anai.2013.06.011
Apter, A. J., Perez, L., Han, X., Ndicu, G., Localio, A., Park, H., Mullen, A. N., Klusaritz, H., Rogers, M., Cidav, Z., Bryant-Stephens, T., Bender, B. G., Reisine, S. T., & Morales, K. H. (2020). Patient Advocates for Low-Income Adults with Moderate to Severe Asthma: A Randomized Clinical Trial. *The journal of allergy and clinical immunology. In practice*, *8*(10), 3466–3473.e11. https://doi.org/10.1016/j.jaip.2020.06.058
Arshi, S., Fallahpour, M., Nabavi, M., Bemanian, M. H., Javad-Mousavi, S. A., Nojomi, M., Esmaeilzadeh, H., Molatefi, R., Rekabi, M., Jalali, F., & Akbarpour, N. (2014). The effects of vitamin D supplementation on airway functions in mild to moderate persistent asthma. *Annals of allergy, asthma & immunology : official publication of the American College of Allergy, Asthma, & Immunology*, *113*(4), 404–409. https://doi.org/10.1016/j.anai.2014.07.005
Badorrek, P., Dick, M., Emmert, L., Schaumann, F., Koch, W., Hecker, H., Murdoch, R., Hohlfeld, J. M., & Krug, N. (2012). Pollen starch granules in bronchial inflammation. *Annals of allergy, asthma & immunology : official publication of the American College of Allergy, Asthma, & Immunology*, *109*(3), 208–214.e6. https://doi.org/10.1016/j.anai.2012.06.019
Baptist, A. P., Hao, W., Song, P. X., Carpenter, L., Steinberg, J., & Cardozo, L. J. (2020). A behavioral intervention can decrease asthma exacerbations in older adults. *Annals of allergy, asthma & immunology : official publication of the American College of Allergy, Asthma, & Immunology*, *124*(3), 248–253.e3. https://doi.org/10.1016/j.anai.2019.12.015
Baris, S., Kiykim, A., Ozen, A., Tulunay, A., Karakoc-Aydiner, E., & Barlan, I. B. (2014). Vitamin D as an adjunct to subcutaneous allergen immunotherapy in asthmatic children sensitized to house dust mite. *Allergy*, *69*(2), 246–253. https://doi.org/10.1111/all.12278
Bensch, G. W., Greos, L. S., Gawchik, S., Kpamegan, E., & Newman, K. B. (2011). Linear growth and bone maturation are unaffected by 1 year of therapy with inhaled flunisolide hydrofluoroalkane in prepubescent children with mild persistent asthma: a randomized, double-blind, placebo-controlled trial. *Annals of allergy, asthma & immunology : official publication of the American College of Allergy, Asthma, & Immunology*, *107*(4), 323–329. https://doi.org/10.1016/j.anai.2011.07.017
Berger, M., de Boer, J. D., Bresser, P., van der Poll, T., Lutter, R., Sterk, P. J., & van der Zee, J. S. (2015). Lipopolysaccharide amplifies eosinophilic inflammation after segmental challenge with house dust mite in asthmatics. *Allergy*, *70*(3), 257–264. https://doi.org/10.1111/all.12544

Berger, W. E., Jacobs, R. L., Amar, N. J., Tantry, S. K., Li, J., & Small, C. J. (2015). Efficacy and safety of beclomethasone dipropionate nasal aerosol in children with perennial allergic rhinitis. *Annals of allergy, asthma & immunology : official publication of the American College of Allergy, Asthma, & Immunology*, *115*(2), 130–136. https://doi.org/10.1016/j.anai.2015.05.012
Berger, W. E., Shah, S., Lieberman, P., Hadley, J., Price, D., Munzel, U., & Bhatia, S. (2014). Long-term, randomized safety study of MP29-02 (a novel intranasal formulation of azelastine hydrochloride and fluticasone propionate in an advanced delivery system) in subjects with chronic rhinitis. *The journal of allergy and clinical immunology. In practice*, *2*(2), 179–185. https://doi.org/10.1016/j.jaip.2013.09.019
Bleecker, E. R., Lötvall, J., O'Byrne, P. M., Woodcock, A., Busse, W. W., Kerwin, E. M., Forth, R., Medley, H. V., Nunn, C., Jacques, L., & Bateman, E. D. (2014). Fluticasone furoate-vilanterol 100-25 mcg compared with fluticasone furoate 100 mcg in asthma: a randomized trial. *The journal of allergy and clinical immunology. In practice*, *2*(5), 553–561. https://doi.org/10.1016/j.jaip.2014.02.010
Boelke, G., Berger, U., Bergmann, K. C., Bindslev-Jensen, C., Bousquet, J., Gildemeister, J., Jutel, M., Pfaar, O., Sehlinger, T., & Zuberbier, T. (2017). Peak nasal inspiratory flow as outcome for provocation studies in allergen exposure chambers: a GA2LEN study. *Clinical and translational allergy*, *7*, 33. https://doi.org/10.1186/s13601-017-0169-4
Bousquet, J., Meltzer, E. O., Couroux, P., Koltun, A., Kopietz, F., Munzel, U., Kuhl, H. C., Nguyen, D. T., Salapatek, A. M., & Price, D. (2018). Onset of Action of the Fixed Combination Intranasal Azelastine-Fluticasone Propionate in an Allergen Exposure Chamber. *The journal of allergy and clinical immunology. In practice*, *6*(5), 1726–1732.e6. https://doi.org/10.1016/j.jaip.2018.01.031
Bożek, A., Kołodziejczyk, K., & Jarząb, J. (2018). Efficacy and safety of birch pollen immunotherapy for local allergic rhinitis. *Annals of allergy, asthma & immunology : official publication of the American College of Allergy, Asthma, & Immunology*, *120*(1), 53–58. https://doi.org/10.1016/j.anai.2017.10.009
Bożek, A., Kołodziejczyk, K., Kozłowska, R., & Canonica, G. W. (2017). Evidence of the efficacy and safety of house dust mite subcutaneous immunotherapy in elderly allergic rhinitis patients: a randomized, double-blind placebo-controlled trial. *Clinical and translational allergy*, *7*, 43. https://doi.org/10.1186/s13601-017-0180-9
Bozek, A., Kolodziejczyk, K., Krajewska-Wojtys, A., & Jarzab, J. (2016). Pre-seasonal, subcutaneous immunotherapy: a double-blinded, placebo-controlled study in elderly patients with an allergy to grass. *Annals of allergy, asthma & immunology : official publication of the American College of Allergy, Asthma, & Immunology*, *116*(2), 156–161. https://doi.org/10.1016/j.anai.2015.12.013
Bozek, A., Starczewska-Dymek, L., & Jarzab, J. (2017). Prolonged effect of allergen sublingual immunotherapy for house dust mites in elderly patients. *Annals of allergy, asthma & immunology : official publication of the American College of Allergy, Asthma, & Immunology*, *119*(1), 77–82. https://doi.org/10.1016/j.anai.2017.05.012
Brown, E. S., Sayed, N., Van Enkevort, E., Kulikova, A., Nakamura, A., Khan, D. A., Ivleva, E. I., Sunderajan, P., Bender, B. G., & Holmes, T. (2018). A Randomized, Double-Blind, Placebo-Controlled Trial of Escitalopram in Patients with Asthma and

Major Depressive Disorder. *The journal of allergy and clinical immunology. In practice*, *6*(5), 1604–1612. https://doi.org/10.1016/j.jaip.2018.01.010
Busse, W. W., Bateman, E. D., O'Byrne, P. M., Lötvall, J., Woodcock, A., Medley, H., Forth, R., & Jacques, L. (2014). Once-daily fluticasone furoate 50 mcg in mild-to-moderate asthma: a 24-week placebo-controlled randomized trial. *Allergy*, *69*(11), 1522–1530. https://doi.org/10.1111/all.12480
Callebaut, I., Vandewalle, E., Hox, V., Bobic, S., Jorissen, M., Stalmans, I., De Vries, A., Scadding, G., & Hellings, P. W. (2012). Nasal corticosteroid treatment reduces substance P levels in tear fluid in allergic rhinoconjunctivitis. *Annals of allergy, asthma & immunology : official publication of the American College of Allergy, Asthma, & Immunology*, *109*(2), 141–146. https://doi.org/10.1016/j.anai.2012.06.008
Cardona, I. D., Kempe, E. E., Lary, C., Ginder, J. H., & Jain, N. (2020). Frequent Versus Infrequent Bathing in Pediatric Atopic Dermatitis: A Randomized Clinical Trial. *The journal of allergy and clinical immunology. In practice*, *8*(3), 1014–1021. https://doi.org/10.1016/j.jaip.2019.10.042
Casale, T. B., Korenblat, P. E., Meltzer, E. O., Yen, K., & Bhatnagar, A. (2011). Nasal carbon dioxide for the symptomatic treatment of perennial allergic rhinitis. *Annals of allergy, asthma & immunology : official publication of the American College of Allergy, Asthma, & Immunology*, *107*(4), 364–370. https://doi.org/10.1016/j.anai.2011.07.014
Choi, S. M., Park, J. E., Li, S. S., Jung, H., Zi, M., Kim, T. H., Jung, S., Kim, A., Shin, M., Sul, J. U., Hong, Z., Jiping, Z., Lee, S., Liyun, H., Kang, K., & Baoyan, L. (2013). A multicenter, randomized, controlled trial testing the effects of acupuncture on allergic rhinitis. *Allergy*, *68*(3), 365–374. https://doi.org/10.1111/all.12053
Davis, B. E., Amakye, D. O., & Cockcroft, D. W. (2015). Airway responsiveness to mannitol 24 h after allergen challenge in atopic asthmatics. *Allergy*, *70*(6), 682–688. https://doi.org/10.1111/all.12601
Devillier, P., Fadel, R., & de Beaumont, O. (2016). House dust mite sublingual immunotherapy is safe in patients with mild-to-moderate, persistent asthma: a clinical trial. *Allergy*, *71*(2), 249–257. https://doi.org/10.1111/all.12791
Didier, A., Malling, H. J., Worm, M., Horak, F., & Sussman, G. L. (2015). Prolonged efficacy of the 300IR 5-grass pollen tablet up to 2 years after treatment cessation, as measured by a recommended daily combined score. *Clinical and translational allergy*, *5*, 12. https://doi.org/10.1186/s13601-015-0057-8
DiMango, E., Serebrisky, D., Narula, S., Shim, C., Keating, C., Sheares, B., Perzanowski, M., Miller, R., DiMango, A., Andrews, H., Merle, D., Liu, X., Calatroni, A., & Kattan, M. (2016). Individualized Household Allergen Intervention Lowers Allergen Level But Not Asthma Medication Use: A Randomized Controlled Trial. *The journal of allergy and clinical immunology. In practice*, *4*(4), 671–679.e4. https://doi.org/10.1016/j.jaip.2016.01.016
Ellis, A. K., Jacobs, R. L., Tenn, M. W., Steacy, L. M., Adams, D. E., Walker, T. J., Togias, A., Ramirez, D. A., Andrews, C. P., Visness, C. M., James, R. L., & Rather, C. G. (2019). Clinical standardization of two controlled allergen challenge facilities: The Environmental Exposure Unit and the Biogenics Research Chamber. *Annals of allergy, asthma & immunology : official publication of the American College of Allergy, Asthma, & Immunology*, *122*(6), 639–646.e2. https://doi.org/10.1016/j.anai.2019.03.007

Ellis, A. K., Tenn, M. W., Steacy, L. M., Adams, D. E., Day, A. G., Walker, T. J., & Nolte, H. (2018). Lack of effect of Timothy grass pollen sublingual immunotherapy tablet on birch pollen-induced allergic rhinoconjunctivitis in an environmental exposure unit. *Annals of allergy, asthma & immunology : official publication of the American College of Allergy, Asthma, & Immunology*, *120*(5), 495–503.e2. https://doi.org/10.1016/j.anai.2018.02.003
Evaristo, K. B., Mendes, F. A. R., Saccomani, M. G., Cukier, A., Carvalho-Pinto, R. M., Rodrigues, M. R., Santaella, D. F., Saraiva-Romanholo, B. M., Martins, M. A., & Carvalho, C. R. F. (2020). Effects of Aerobic Training Versus Breathing Exercises on Asthma Control: A Randomized Trial. *The journal of allergy and clinical immunology. In practice*, *8*(9), 2989–2996.e4. https://doi.org/10.1016/j.jaip.2020.06.042
Fujishima, H., Ohashi, Y., & Takamura, E. (2014). Efficacy of epinastine hydrochloride ophthalmic solution in allergic conjunctivitis by conjunctival cedar pollen allergen challenge. *Annals of allergy, asthma & immunology : official publication of the American College of Allergy, Asthma, & Immunology*, *113*(4), 476–481. https://doi.org/10.1016/j.anai.2014.07.007
Goujon, C., Viguier, M., Staumont-Sallé, D., Bernier, C., Guillet, G., Lahfa, M., Ferrier Le Bouedec, M. C., Cambazard, F., Bottigioli, D., Grande, S., Dahel, K., Bérard, F., Rabilloud, M., Mercier, C., & Nicolas, J. F. (2018). Methotrexate Versus Cyclosporine in Adults with Moderate-to-Severe Atopic Dermatitis: A Phase III Randomized Noninferiority Trial. *The journal of allergy and clinical immunology. In practice*, *6*(2), 562–569.e3. https://doi.org/10.1016/j.jaip.2017.07.007
Gross, G. N., Berman, G., Amar, N. J., Caracta, C. F., & Tantry, S. K. (2019). Efficacy and safety of olopatadine-mometasone combination nasal spray for the treatment of seasonal allergic rhinitis. *Annals of allergy, asthma & immunology : official publication of the American College of Allergy, Asthma, & Immunology*, *122*(6), 630–638.e3. https://doi.org/10.1016/j.anai.2019.03.017
Hampel, F. C., Jr, Nayak, N. A., Segall, N., Small, C. J., Li, J., & Tantry, S. K. (2015). No hypothalamic-pituitary-adrenal function effect with beclomethasone dipropionate nasal aerosol, based on 24-hour serum cortisol in pediatric allergic rhinitis. *Annals of allergy, asthma & immunology : official publication of the American College of Allergy, Asthma, & Immunology*, *115*(2), 137–142. https://doi.org/10.1016/j.anai.2015.05.019
Hendeles, L., Khan, Y. R., Shuster, J. J., Chesrown, S. E., & Abu-Hasan, M. (2015). Omalizumab therapy for asthma patients with poor adherence to inhaled corticosteroid therapy. *Annals of allergy, asthma & immunology : official publication of the American College of Allergy, Asthma, & Immunology*, *114*(1), 58–62.e2. https://doi.org/10.1016/j.anai.2014.10.012
Higaki, T., Okano, M., Makihara, S., Fujiwara, T., Haruna, T., Noda, Y., Kariya, S., & Nishizaki, K. (2012). Early interventional treatment with intranasal corticosteroids compared with postonset treatment in pollinosis. *Annals of allergy, asthma & immunology : official publication of the American College of Allergy, Asthma, & Immunology*, *109*(6), 458–464. https://doi.org/10.1016/j.anai.2012.08.016
Hoshino, M., Akitsu, K., & Kubota, K. (2019). Effect of Sublingual Immunotherapy on Airway Inflammation and Airway Wall Thickness in Allergic Asthma. *The journal of allergy and clinical immunology. In practice*, *7*(8), 2804–2811. https://doi.org/10.1016/j.jaip.2019.06.003

Hoshino, M., Akitsu, K., Kubota, K., & Ohtawa, J. (2021). Serum Periostin as a Biomarker for Predicting Clinical Response to House Dust Mite Sublingual Immunotherapy in Allergic Rhinitis. *The journal of allergy and clinical immunology. In practice*, *9*(5), 1864–1870. https://doi.org/10.1016/j.jaip.2020.11.046
Ihara, F., Sakurai, D., Yonekura, S., Iinuma, T., Yagi, R., Sakurai, T., Ito, T., Matsuura, A., Morimoto, Y., Arai, T., Suzuki, S., Katayama, K., Nakayama, T., & Okamoto, Y. (2018). Identification of specifically reduced Th2 cell subsets in allergic rhinitis patients after sublingual immunotherapy. *Allergy*, *73*(9), 1823–1832. https://doi.org/10.1111/all.13436
Janeczek, K., Emeryk, A., Rachel, M., Duma, D., Zimmer, Ł., & Poleszak, E. (2021). Polyvalent Mechanical Bacterial Lysate Administration Improves the Clinical Course of Grass Pollen-Induced Allergic Rhinitis in Children: A Randomized Controlled Trial. *The journal of allergy and clinical immunology. In practice*, *9*(1), 453–462. https://doi.org/10.1016/j.jaip.2020.08.025
Kerwin, E. M., Preece, A., Brintziki, D., Collison, K. A., & Sharma, R. (2019). ELLIPTA Dry Powder Versus Metered-Dose Inhalers in an Optimized Clinical Trial Setting. *The journal of allergy and clinical immunology. In practice*, *7*(6), 1843–1849. https://doi.org/10.1016/j.jaip.2019.02.023
Khusial, R. J., Honkoop, P. J., Usmani, O., Soares, M., Simpson, A., Biddiscombe, M., Meah, S., Bonini, M., Lalas, A., Polychronidou, E., Koopmans, J. G., Moustakas, K., Snoeck-Stroband, J. B., Ortmann, S., Votis, K., Tzovaras, D., Chung, K. F., Fowler, S., Sont, J. K., & myAirCoach study group (2020). Effectiveness of myAirCoach: A mHealth Self-Management System in Asthma. *The journal of allergy and clinical immunology. In practice*, *8*(6), 1972–1979.e8. https://doi.org/10.1016/j.jaip.2020.02.018
Kim, S. H., Lee, T., Jang, A. S., Park, C. S., Jung, J. W., Kim, M. H., Kwon, J. W., Moon, J. Y., Yang, M. S., Lee, J., Choi, J. H., Shin, Y. S., Kim, H. K., Kim, S., Kim, J. H., Lee, S. Y., Nam, Y. H., Kim, S. H., Kim, T. B., & Cohort for Reality and Evolution of Adult Asthma (COREA) Study Group (2021). Pragmatic Randomized Controlled Trial for Stepping Down Asthma Controller Treatment in Patients Controlled with Low-Dose Inhaled Corticosteroid and Long-Acting β2-Agonist: Step-Down of Intervention and Grade in Moderate Asthma Study. *The journal of allergy and clinical immunology. In practice*, *9*(10), 3638–3646.e3. https://doi.org/10.1016/j.jaip.2021.04.042
Klimek, L., Sperl, A., van Twuijver, E., van Ree, R., Kleinjans, H., Boot, J. D., & Pfaar, O. (2014). A prospective study comparing the efficacy and safety of two sublingual birch allergen preparations. *Clinical and translational allergy*, *4*, 23. https://doi.org/10.1186/2045-7022-4-23
Kopac, P., Rudin, M., Gentinetta, T., Gerber, R., Pichler, C.h, Hausmann, O., Schnyder, B., & Pichler, W. J. (2012). Continuous apple consumption induces oral tolerance in birch-pollen-associated apple allergy. *Allergy*, *67*(2), 280–285. https://doi.org/10.1111/j.1398-9995.2011.02744.x
Kortekaas Krohn, I., Callebaut, I., Alpizar, Y. A., Steelant, B., Van Gerven, L., Skov, P. S., Kasran, A., Talavera, K., Wouters, M. M., Ceuppens, J. L., Seys, S. F., & Hellings, P. W. (2018). MP29-02 reduces nasal hyperreactivity and nasal mediators in patients with house dust mite-allergic rhinitis. *Allergy*, *73*(5), 1084–1093. https://doi.org/10.1111/all.13349

Kralimarkova, T. Z., Popov, T. A., Staevska, M., Mincheva, R., Lazarova, C., Racheva, R., Mustakov, T. B., Filipova, V., Koleva, M., Bacheva, K., & Dimitrov, V. D. (2014). Objective approach for fending off the sublingual immunotherapy placebo effect in subjects with pollenosis: double-blinded, placebo-controlled trial. *Annals of allergy, asthma & immunology : official publication of the American College of Allergy, Asthma, & Immunology*, *113*(1), 108–113. https://doi.org/10.1016/j.anai.2014.03.019
LaForce, C., Journeay, G. E., Miller, S. D., Silvey, M. J., Wu, W., Lee, L. A., & Chylack, L. T., Jr (2013). Ocular safety of fluticasone furoate nasal spray in patients with perennial allergic rhinitis: a 2-year study. *Annals of allergy, asthma & immunology : official publication of the American College of Allergy, Asthma, & Immunology*, *111*(1), 45–50. https://doi.org/10.1016/j.anai.2013.04.013
Laidlaw, T. M., Bachert, C., Amin, N., Desrosiers, M., Hellings, P. W., Mullol, J., Maspero, J. F., Gevaert, P., Zhang, M., Mao, X., Khan, A. H., Kamat, S., Patel, N., Graham, N. M. H., Ruddy, M., Staudinger, H., & Mannent, L. P. (2021). Dupilumab improves upper and lower airway disease control in chronic rhinosinusitis with nasal polyps and asthma. *Annals of allergy, asthma & immunology : official publication of the American College of Allergy, Asthma, & Immunology*, *126*(5), 584–592.e1. https://doi.org/10.1016/j.anai.2021.01.012
Lee, J. H., Kim, W. S., Kim, H., & Hahn, Y. S. (2013). Increased cow's milk protein-specific IgG4 levels after oral desensitization in 7- to 12-month-old infants. *Annals of allergy, asthma & immunology : official publication of the American College of Allergy, Asthma, & Immunology*, *111*(6), 523–528. https://doi.org/10.1016/j.anai.2013.09.001
Lee, L. A., Sterling, R., Máspero, J., Clements, D., Ellsworth, A., & Pedersen, S. (2014). Growth velocity reduced with once-daily fluticasone furoate nasal spray in prepubescent children with perennial allergic rhinitis. *The journal of allergy and clinical immunology. In practice*, *2*(4), 421–427. https://doi.org/10.1016/j.jaip.2014.04.008
Lipworth, B. J., Short, P., Burns, P., & Nair, A. (2012). Effects of intranasal salmeterol and fluticasone given alone and in combination in persistent allergic rhinitis. *Annals of allergy, asthma & immunology : official publication of the American College of Allergy, Asthma, & Immunology*, *108*(1), 54–59. https://doi.org/10.1016/j.anai.2011.10.001
Lötvall, J., Palmqvist, M., & Arvidsson, P. (2011). Comparing the effects of two inhaled glucocorticoids on allergen-induced bronchoconstriction and markers of systemic effects, a randomised cross-over double-blind study. *Clinical and translational allergy*, *1*(1), 12. https://doi.org/10.1186/2045-7022-1-12
Lou, H., Huang, Y., Ouyang, Y., Zhang, Y., Xi, L., Chu, X., Wang, Y., Wang, C., & Zhang, L. (2020). Artemisia annua-sublingual immunotherapy for seasonal allergic rhinitis: A randomized controlled trial. *Allergy*, *75*(8), 2026–2036. https://doi.org/10.1111/all.14218
Maloney, J., Bernstein, D. I., Nelson, H., Creticos, P., Hébert, J., Noonan, M., Skoner, D., Zhou, Y., Kaur, A., & Nolte, H. (2014). Efficacy and safety of grass sublingual immunotherapy tablet, MK-7243: a large randomized controlled trial. *Annals of allergy, asthma & immunology : official publication of the American College of Allergy, Asthma, & Immunology*, *112*(2), 146–153.e2. https://doi.org/10.1016/j.anai.2013.11.018
Maspero, J., Backer, V., Yao, R., Staudinger, H., & Teper, A. (2013). Effects of mometasone, fluticasone, and montelukast on bone mineral density in adults with

asthma. *The journal of allergy and clinical immunology. In practice*, *1*(6), 649–55.e1. https://doi.org/10.1016/j.jaip.2013.07.011
Masuyama, K., Okamoto, Y., Okamiya, K., Azuma, R., Fujinami, T., Riis, B., Ohashi-Doi, K., Natsui, K., Imai, T., & Okubo, K. (2018). Efficacy and safety of SQ house dust mite sublingual immunotherapy-tablet in Japanese children. *Allergy*, *73*(12), 2352–2363. https://doi.org/10.1111/all.13544
Matsumoto, M., Ebata, T., Hirooka, J., Hosoya, R., Inoue, N., Itami, S., Tsuji, K., Yaginuma, T., Muramatsu, K., Nakamura, A., Fujita, A., & Nagakura, T. (2014). Antipruritic effects of the probiotic strain LKM512 in adults with atopic dermatitis. *Annals of allergy, asthma & immunology : official publication of the American College of Allergy, Asthma, & Immunology*, *113*(2), 209–216.e7. https://doi.org/10.1016/j.anai.2014.05.002
McDonald, J. L., Smith, P. K., Smith, C. A., Changli Xue, C., Golianu, B., Cripps, A. W., & Mucosal Immunology Research Group (2016). Effect of acupuncture on house dust mite specific IgE, substance P, and symptoms in persistent allergic rhinitis. *Annals of allergy, asthma & immunology : official publication of the American College of Allergy, Asthma, & Immunology*, *116*(6), 497–505. https://doi.org/10.1016/j.anai.2016.04.002
Meltzer, E. O., Ratner, P. H., & McGraw, T. (2016). Phenylephrine hydrochloride modified-release tablets for nasal congestion: a randomized, placebo-controlled trial in allergic rhinitis patients. *Annals of allergy, asthma & immunology : official publication of the American College of Allergy, Asthma, & Immunology*, *116*(1), 66–71. https://doi.org/10.1016/j.anai.2015.10.022
Merchant, R. K., Inamdar, R., & Quade, R. C. (2016). Effectiveness of Population Health Management Using the Propeller Health Asthma Platform: A Randomized Clinical Trial. *The journal of allergy and clinical immunology. In practice*, *4*(3), 455–463. https://doi.org/10.1016/j.jaip.2015.11.022
Meyer, W., Narkus, A., Salapatek, A. M., & Häfner, D. (2013). Double-blind, placebo-controlled, dose-ranging study of new recombinant hypoallergenic Bet v 1 in an environmental exposure chamber. *Allergy*, *68*(6), 724–731. https://doi.org/10.1111/all.12148
Morgan, M., Cooke, A., Rogers, L., Adams-Huet, B., & Khan, D. A. (2014). Double-blind placebo-controlled trial of dapsone in antihistamine refractory chronic idiopathic urticaria. *The journal of allergy and clinical immunology. In practice*, *2*(5), 601–606. https://doi.org/10.1016/j.jaip.2014.06.004
Morphew, T., Shin, H. W., Marchese, S., Pires-Barracosa, N., & Galant, S. P. (2019). Phenotypes favoring fractional exhaled nitric oxide discordance vs guideline-based uncontrolled asthma. *Annals of allergy, asthma & immunology : official publication of the American College of Allergy, Asthma, & Immunology*, *123*(2), 193–200. https://doi.org/10.1016/j.anai.2019.05.008
Mösges, R., Bachert, C., Panzner, P., Calderon, M. A., Haazen, L., Pirotton, S., Wathelet, N., Durham, S. R., Bonny, M. A., Legon, T., von Frenckell, R., Pfaar, O., & Shamji, M. H. (2018). Short course of grass allergen peptides immunotherapy over 3 weeks reduces seasonal symptoms in allergic rhinoconjunctivitis with/without asthma: A randomized, multicenter, double-blind, placebo-controlled trial. *Allergy*, *73*(9), 1842–1850. https://doi.org/10.1111/all.13433

Mosnaim, G., Li, H., Martin, M., Richardson, D., Belice, P. J., Avery, E., Ryan, N., Bender, B., & Powell, L. (2013). The impact of peer support and mp3 messaging on adherence to inhaled corticosteroids in minority adolescents with asthma: a randomized, controlled trial. *The journal of allergy and clinical immunology. In practice*, *1*(5), 485–493. https://doi.org/10.1016/j.jaip.2013.06.010
Naji, N., Keung, E., Beaudin, S., Kane, J., Killian, K. J., & Gauvreau, G. M. (2013). The effects of particle size on measurement of airway hyperresponsiveness to methacholine. *Annals of allergy, asthma & immunology : official publication of the American College of Allergy, Asthma, & Immunology*, *110*(5), 359–363. https://doi.org/10.1016/j.anai.2013.02.014
Nakaji, H., Petrova, G., Matsumoto, H., Iwata, T., Ito, I., Oguma, T., Inoue, H., Tajiri, T., Nagasaki, T., Kanemitsu, Y., Niimi, A., & Mishima, M. (2013). Effects of 24-week add-on treatment with ciclesonide and montelukast on small airways inflammation in asthma. *Annals of allergy, asthma & immunology : official publication of the American College of Allergy, Asthma, & Immunology*, *110*(3), 198–203.e3. https://doi.org/10.1016/j.anai.2012.12.016
Ng, C. C., Romaikin, D., Steacy, L. M., Stevens, D. A., Walker, T. J., Adams, D. E., & Ellis, A. K. (2021). Comparative nasal airflow with loratadine-pseudoephedrine and fluticasone nasal spray for allergic rhinitis. *Annals of allergy, asthma & immunology : official publication of the American College of Allergy, Asthma, & Immunology*, *127*(3), 342–348.e2. https://doi.org/10.1016/j.anai.2021.05.001
Nolte, H., Bernstein, D. I., Nelson, H. S., Ellis, A. K., Kleine-Tebbe, J., & Lu, S. (2020). Efficacy and Safety of Ragweed SLIT-Tablet in Children with Allergic Rhinoconjunctivitis in a Randomized, Placebo-Controlled Trial. *The journal of allergy and clinical immunology. In practice*, *8*(7), 2322–2331.e5. https://doi.org/10.1016/j.jaip.2020.03.041
O'Dwyer, S., Greene, G., MacHale, E., Cushen, B., Sulaiman, I., Boland, F., Bosnic-Anticevich, S., Mokoka, M. C., Reilly, R. B., Taylor, T., Ryder, S. A., & Costello, R. W. (2020). Personalized Biofeedback on Inhaler Adherence and Technique by Community Pharmacists: A Cluster Randomized Clinical Trial. *The journal of allergy and clinical immunology. In practice*, *8*(2), 635–644. https://doi.org/10.1016/j.jaip.2019.09.008
Okamoto, Y., Fujieda, S., Okano, M., Yoshida, Y., Kakudo, S., & Masuyama, K. (2017). House dust mite sublingual tablet is effective and safe in patients with allergic rhinitis. *Allergy*, *72*(3), 435–443. https://doi.org/10.1111/all.12996
Okubo, K., Okano, M., Sato, N., Tamaki, Y., Suzuki, H., Uddin, A., & Fogel, R. (2020). Add-On Omalizumab for Inadequately Controlled Severe Pollinosis Despite Standard-of-Care: A Randomized Study. *The journal of allergy and clinical immunology. In practice*, *8*(9), 3130–3140.e2. https://doi.org/10.1016/j.jaip.2020.04.068
Oliver, A., Quinn, D., Goldfrad, C., van Hecke, B., Ayer, J., & Boyce, M. (2012). Combined fluticasone furoate/vilanterol reduces decline in lung function following inhaled allergen 23 h after dosing in adult asthma: a randomised, controlled trial. *Clinical and translational allergy*, *2*(1), 11. https://doi.org/10.1186/2045-7022-2-11
Paggiaro, P., Halpin, D. M., Buhl, R., Engel, M., Zubek, V. B., Blahova, Z., Moroni-Zentgraf, P., & Pizzichini, E. (2016). The Effect of Tiotropium in Symptomatic Asthma Despite Low- to Medium-Dose Inhaled Corticosteroids: A Randomized Controlled

Trial. *The journal of allergy and clinical immunology. In practice*, *4*(1), 104–13.e2. https://doi.org/10.1016/j.jaip.2015.08.017
Park, S. Y., Kim, S., Kim, J. H., Kim, S. H., Lee, T., Yoon, S. Y., Kim, M. H., Moon, J. Y., Yang, M. S., Jung, J. W., Kim, J. H., Choi, J. H., Park, C. S., Kim, S., Lee, J., Kwon, J. W., Hur, G. Y., Kim, S. H., Kim, H. K., Shin, Y. S., … Cohort for Reality and Evolution of Adult Asthma in Korea (COREA) Study Group (2021). A Randomized, Noninferiority Trial Comparing ICS + LABA with ICS + LABA + LAMA in Asthma-COPD Overlap (ACO) Treatment: The ACO Treatment with Optimal Medications (ATOMIC) Study. *The journal of allergy and clinical immunology. In practice*, *9*(3), 1304–1311.e2. https://doi.org/10.1016/j.jaip.2020.09.066
Patel, M. R., Song, P. X., Sanders, G., Nelson, B., Kaltsas, E., Thomas, L. J., Janevic, M. R., Hafeez, K., Wang, W., Wilkin, M., Johnson, T. R., & Brown, R. W. (2017). A randomized clinical trial of a culturally responsive intervention for African American women with asthma. *Annals of allergy, asthma & immunology : official publication of the American College of Allergy, Asthma, & Immunology*, *118*(2), 212–219. https://doi.org/10.1016/j.anai.2016.11.016
Patel, M., Pilcher, J., Travers, J., Perrin, K., Shaw, D., Black, P., Weatherall, M., & Beasley, R. (2013). Use of metered-dose inhaler electronic monitoring in a real-world asthma randomized controlled trial. *The journal of allergy and clinical immunology. In practice*, *1*(1), 83–91. https://doi.org/10.1016/j.jaip.2012.08.004
Pearlman, D. S., Eckerwall, G., McLaren, J., Lamarca, R., Puu, M., Gilbert, I., Jorup, C., Sandin, K., & Lanz, M. J. (2017). Efficacy and safety of budesonide/formoterol pMDI vs budesonide pMDI in asthmatic children (6-<12 years). *Annals of allergy, asthma & immunology : official publication of the American College of Allergy, Asthma, & Immunology*, *118*(4), 489–499.e1. https://doi.org/10.1016/j.anai.2017.01.020
Perrin, Y., Nutten, S., Audran, R., Berger, B., Bibiloni, R., Wassenberg, J., Barbier, N., Aubert, V., Moulin, J., Singh, A., Magliola, C., Mercenier, A., & Spertini, F. (2014). Comparison of two oral probiotic preparations in a randomized crossover trial highlights a potentially beneficial effect of Lactobacillus paracasei NCC2461 in patients with allergic rhinitis. *Clinical and translational allergy*, *4*(1), 1. https://doi.org/10.1186/2045-7022-4-1
Perry, T. T., Halterman, J. S., Brown, R. H., Luo, C., Randle, S. M., Hunter, C. R., & Rettiganti, M. (2018). Results of an asthma education program delivered via telemedicine in rural schools. *Annals of allergy, asthma & immunology : official publication of the American College of Allergy, Asthma, & Immunology*, *120*(4), 401–408. https://doi.org/10.1016/j.anai.2018.02.013
Pfaar, O., Biedermann, T., Klimek, L., Sager, A., & Robinson, D. S. (2013). Depigmented-polymerized mixed grass/birch pollen extract immunotherapy is effective in polysensitized patients. *Allergy*, *68*(10), 1306–1313. https://doi.org/10.1111/all.12219
Pfaar, O., Urry, Z., Robinson, D. S., Sager, A., Richards, D., Hawrylowicz, C. M., Bräutigam, M., & Klimek, L. (2012). A randomized placebo-controlled trial of rush preseasonal depigmented polymerized grass pollen immunotherapy. *Allergy*, *67*(2), 272–279. https://doi.org/10.1111/j.1398-9995.2011.02736.x
Pfab, F., Kirchner, M. T., Huss-Marp, J., Schuster, T., Schalock, P. C., Fuqin, J., Athanasiadis, G. I., Behrendt, H., Ring, J., Darsow, U., & Napadow, V. (2012). Acupuncture compared with oral antihistamine for type I hypersensitivity itch and skin

response in adults with atopic dermatitis: a patient- and examiner-blinded, randomized, placebo-controlled, crossover trial. *Allergy*, *67*(4), 566–573. https://doi.org/10.1111/j.1398-9995.2012.02789.x

Potter, P. C., Baker, S., Fenemore, B., & Nurse, B. (2015). Clinical and cytokine responses to house dust mite sublingual immunotherapy. *Annals of allergy, asthma & immunology : official publication of the American College of Allergy, Asthma, & Immunology*, *114*(4), 327–334. https://doi.org/10.1016/j.anai.2014.12.015

Ratner, P. H., Miller, S. D., Hampel, F. C., Jr, Melchior, A., Dunbar, S. A., & Tantry, S. K. (2012). Once-daily treatment with beclomethasone dipropionate nasal aerosol does not affect hypothalamic-pituitary-adrenal axis function. *Annals of allergy, asthma & immunology : official publication of the American College of Allergy, Asthma, & Immunology*, *109*(5), 336–341. https://doi.org/10.1016/j.anai.2012.08.005

Ratner, P., Van Bavel, J., Mohar, D., Jacobs, R. L., Hampel, F., Howland, W., & Karwal, R. (2015). Efficacy of daily intranasal fluticasone propionate on ocular symptoms associated with seasonal allergic rhinitis. *Annals of allergy, asthma & immunology : official publication of the American College of Allergy, Asthma, & Immunology*, *114*(2), 141–147. https://doi.org/10.1016/j.anai.2014.11.012

Rogers, L., Sugar, E. A., Blake, K., Castro, M., Dimango, E., Hanania, N. A., Happel, K. I., Peters, S. P., Reibman, J., Saams, J., Teague, W. G., Wise, R. A., Holbrook, J. T., & American Lung Association Airways Clinical Research Centers (2018). Step-Down Therapy for Asthma Well Controlled on Inhaled Corticosteroid and Long-Acting Beta-Agonist: A Randomized Clinical Trial. *The journal of allergy and clinical immunology. In practice*, *6*(2), 633–643.e1. https://doi.org/10.1016/j.jaip.2017.07.030

Rorie, A., Goldner, W. S., Lyden, E., & Poole, J. A. (2014). Beneficial role for supplemental vitamin D3 treatment in chronic urticaria: a randomized study. *Annals of allergy, asthma & immunology : official publication of the American College of Allergy, Asthma, & Immunology*, *112*(4), 376–382. https://doi.org/10.1016/j.anai.2014.01.010

Sánchez, J., Zakzuk, J., & Cardona, R. (2018). Evaluation of a Guidelines-Based Approach to the Treatment of Chronic Spontaneous Urticaria. *The journal of allergy and clinical immunology. In practice*, *6*(1), 177–182.e1. https://doi.org/10.1016/j.jaip.2017.06.002

Şanlıtürk, D., & Ayaz-Alkaya, S. (2021). The Effect of a Theory of Planned Behavior Education Program on Asthma Control and Medication Adherence: A Randomized Controlled Trial. *The journal of allergy and clinical immunology. In practice*, *9*(9), 3371–3379. https://doi.org/10.1016/j.jaip.2021.03.060

Schmid, J. M., Würtzen, P. A., Siddhuraj, P., Jogdand, P., Petersen, C. G., Dahl, R., Erjefält, J. S., & Hoffmann, H. J. (2021). Basophil sensitivity reflects long-term clinical outcome of subcutaneous immunotherapy in grass pollen-allergic patients. *Allergy*, *76*(5), 1528–1538. https://doi.org/10.1111/all.14264

Scott, H. A., Latham, J. R., Callister, R., Pretto, J. J., Baines, K., Saltos, N., Upham, J. W., & Wood, L. G. (2015). Acute exercise is associated with reduced exhaled nitric oxide in physically inactive adults with asthma. *Annals of allergy, asthma & immunology : official publication of the American College of Allergy, Asthma, & Immunology*, *114*(6), 470–479. https://doi.org/10.1016/j.anai.2015.04.002

Shamji, M. H., Ljørring, C., Francis, J. N., Calderon, M. A., Larché, M., Kimber, I., Frew, A. J., Ipsen, H., Lund, K., Würtzen, P. A., & Durham, S. R. (2012). Functional

rather than immunoreactive levels of IgG4 correlate closely with clinical response to grass pollen immunotherapy. *Allergy*, *67*(2), 217–226. https://doi.org/10.1111/j.1398-9995.2011.02745.x

Smith, H., Horney, D., Goubet, S., Jones, C., Raza, A., White, P., & Frew, A. (2015). Pragmatic randomized controlled trial of a structured allergy intervention for adults with asthma and rhinitis in general practice. *Allergy*, *70*(2), 203–211. https://doi.org/10.1111/all.12550

Staubach, P., Metz, M., Chapman-Rothe, N., Sieder, C., Bräutigam, M., Canvin, J., & Maurer, M. (2016). Effect of omalizumab on angioedema in H1 -antihistamine-resistant chronic spontaneous urticaria patients: results from X-ACT, a randomized controlled trial. *Allergy*, *71*(8), 1135–1144. https://doi.org/10.1111/all.12870

Stelmach, I., Kaluzińska-Parzyszek, I., Jerzynska, J., Stelmach, P., Stelmach, W., & Majak, P. (2012). Comparative effect of pre-coseasonal and continuous grass sublingual immunotherapy in children. *Allergy*, *67*(3), 312–320. https://doi.org/10.1111/j.1398-9995.2011.02758.x

Stewart, S. L., Martin, A. L., Davis, B. E., & Cockcroft, D. W. (2012). Salbutamol tolerance to bronchoprotection: course of onset. *Annals of allergy, asthma & immunology : official publication of the American College of Allergy, Asthma, & Immunology*, *109*(6), 454–457. https://doi.org/10.1016/j.anai.2012.08.003

Storms, W. W., Segall, N., Mansfield, L. E., Amar, N. J., Kelley, L., Ding, Y., & Tantry, S. K. (2013). Efficacy and safety of beclomethasone dipropionate nasal aerosol in pediatric patients with seasonal allergic rhinitis. *Annals of allergy, asthma & immunology : official publication of the American College of Allergy, Asthma, & Immunology*, *111*(5), 408–414.e1. https://doi.org/10.1016/j.anai.2013.07.033

Stukus, D. R., Farooqui, N., Strothman, K., Ryan, K., Zhao, S., Stevens, J. H., & Cohen, D. M. (2018). Real-world evaluation of a mobile health application in children with asthma. *Annals of allergy, asthma & immunology : official publication of the American College of Allergy, Asthma, & Immunology*, *120*(4), 395–400.e1. https://doi.org/10.1016/j.anai.2018.02.006

Sudini, K., Diette, G. B., Breysse, P. N., McCormack, M. C., Bull, D., Biswal, S., Zhai, S., Brereton, N., Peng, R. D., & Matsui, E. C. (2016). A Randomized Controlled Trial of the Effect of Broccoli Sprouts on Antioxidant Gene Expression and Airway Inflammation in Asthmatics. *The journal of allergy and clinical immunology. In practice*, *4*(5), 932–940. https://doi.org/10.1016/j.jaip.2016.03.012

Sumino, K., Bacharier, L. B., Taylor, J., Chadwick-Mansker, K., Curtis, V., Nash, A., Jackson-Triggs, S., Moen, J., Schechtman, K. B., Garbutt, J., & Castro, M. (2020). A Pragmatic Trial of Symptom-Based Inhaled Corticosteroid Use in African-American Children with Mild Asthma. *The journal of allergy and clinical immunology. In practice*, *8*(1), 176–185.e2. https://doi.org/10.1016/j.jaip.2019.06.030

Sussman, G., Hébert, J., Gulliver, W., Lynde, C., Yang, W. H., Papp, K., Gooderham, M., Chambenoit, O., Khalil, S., DeTakacsy, F., Vieira, A., & Rihakova, L. (2020). Omalizumab Re-Treatment and Step-Up in Patients with Chronic Spontaneous Urticaria: OPTIMA Trial. *The journal of allergy and clinical immunology. In practice*, *8*(7), 2372–2378.e5. https://doi.org/10.1016/j.jaip.2020.03.022

Suzuki, T., Okamoto, Y., Yonekura, S., Okuma, Y., Sakurai, T., & Sakurai, D. (2016). Characteristics of laryngeal symptoms induced in patients with allergic rhinitis in an

environmental challenge chamber. *Annals of allergy, asthma & immunology : official publication of the American College of Allergy, Asthma, & Immunology*, *116*(6), 491–496. https://doi.org/10.1016/j.anai.2016.03.011

Syk, J., Malinovschi, A., Johansson, G., Undén, A. L., Andreasson, A., Lekander, M., & Alving, K. (2013). Anti-inflammatory treatment of atopic asthma guided by exhaled nitric oxide: a randomized, controlled trial. *The journal of allergy and clinical immunology. In practice*, *1*(6), 639–48.e488. https://doi.org/10.1016/j.jaip.2013.07.013

Tachimoto, H., Mezawa, H., Segawa, T., Akiyama, N., Ida, H., & Urashima, M. (2016). Improved control of childhood asthma with low-dose, short-term vitamin D supplementation: a randomized, double-blind, placebo-controlled trial. *Allergy*, *71*(7), 1001–1009. https://doi.org/10.1111/all.12856

Thongngarm, T., Wongsa, C., Phinyo, P., Assanasen, P., Tantilipikorn, P., & Sompornrattanaphan, M. (2021). As-Needed Versus Regular Use of Fluticasone Furoate Nasal Spray in Patients with Moderate to Severe, Persistent, Perennial Allergic Rhinitis: A Randomized Controlled Trial. *The journal of allergy and clinical immunology. In practice*, *9*(3), 1365–1373.e6. https://doi.org/10.1016/j.jaip.2020.09.057

Toennesen, L. L., Meteran, H., Hostrup, M., Wium Geiker, N. R., Jensen, C. B., Porsbjerg, C., Astrup, A., Bangsbo, J., Parker, D., & Backer, V. (2018). Effects of Exercise and Diet in Nonobese Asthma Patients-A Randomized Controlled Trial. *The journal of allergy and clinical immunology. In practice*, *6*(3), 803–811. https://doi.org/10.1016/j.jaip.2017.09.028

Tzanetos, D. B., Fahrenholz, J. M., Scott, T., & Buchholz, K. (2011). Comparison of the sedating effects of levocetirizine and cetirizine: a randomized, double-blind, placebo-controlled trial. *Annals of allergy, asthma & immunology : official publication of the American College of Allergy, Asthma, & Immunology*, *107*(6), 517–522. https://doi.org/10.1016/j.anai.2011.08.012

Usmani, O. S., Kemppinen, A., Gardener, E., Thomas, V., Konduru, P. R., Callan, C., McLoughlin, A., Woodhead, V., Brady, A., Juniper, E. F., Barnes, P. J., & Price, D. (2017). A Randomized Pragmatic Trial of Changing to and Stepping Down Fluticasone/Formoterol in Asthma. *The journal of allergy and clinical immunology. In practice*, *5*(5), 1378–1387.e5. https://doi.org/10.1016/j.jaip.2017.02.006

van de Pol, M. A., Lutter, R., van Ree, R., & van der Zee, J. S. (2012). Increase in allergen-specific IgE and ex vivo Th2 responses after a single bronchial challenge with house dust mite in allergic asthmatics. *Allergy*, *67*(1), 67–73. https://doi.org/10.1111/j.1398-9995.2011.02722.x

Van Overtvelt, L., Baron-Bodo, V., Horiot, S., Moussu, H., Ricarte, C., Horak, F., Zieglmayer, P., Zieglmayer, R., Montagut, A., Galvain, S., de Beaumont, O., Le Gall, M., & Moingeon, P. (2011). Changes in basophil activation during grass-pollen sublingual immunotherapy do not correlate with clinical efficacy. *Allergy*, *66*(12), 1530–1537. https://doi.org/10.1111/j.1398-9995.2011.02696.x

Videler, W. J., Badia, L., Harvey, R. J., Gane, S., Georgalas, C., van der Meulen, F. W., Menger, D. J., Lehtonen, M. T., Toppila-Salmi, S. K., Vento, S. I., Hytönen, M., Hellings, P. W., Kalogjera, L., Lund, V. J., Scadding, G., Mullol, J., & Fokkens, W. J. (2011). Lack of efficacy of long-term, low-dose azithromycin in chronic rhinosinusitis: a randomized controlled trial. *Allergy*, *66*(11), 1457–1468. https://doi.org/10.1111/j.1398-9995.2011.02693.x

Vuurman, E. F., Vuurman, L. L., Lutgens, I., & Kremer, B. (2014). Allergic rhinitis is a risk factor for traffic safety. *Allergy*, *69*(7), 906–912. https://doi.org/10.1111/all.12418
Wang, L., Yin, J., Fadel, R., Montagut, A., de Beaumont, O., & Devillier, P. (2014). House dust mite sublingual immunotherapy is safe and appears to be effective in moderate, persistent asthma. *Allergy*, *69*(9), 1181–1188. https://doi.org/10.1111/all.12188
Wartna, J. B., Bohnen, A. M., Elshout, G., Pijnenburg, M. W., Pols, D. H., Gerth van Wijk, R. R., & Bindels, P. J. (2017). Symptomatic treatment of pollen-related allergic rhinoconjunctivitis in children: randomized controlled trial. *Allergy*, *72*(4), 636–644. https://doi.org/10.1111/all.13056
Westbury, G. L. M., Blais, C. M., Davis, B. E., & Cockcroft, D. W. (2018). Bronchoprotective effect of vilanterol against methacholine-induced bronchoconstriction in mild asthmatics: A randomized three-way crossover study. *Annals of allergy, asthma & immunology : official publication of the American College of Allergy, Asthma, & Immunology*, *121*(3), 328–332. https://doi.org/10.1016/j.anai.2018.07.005
Worm, M., Rak, S., de Blay, F., Malling, H. J., Melac, M., Cadic, V., & Zeldin, R. K. (2014). Sustained efficacy and safety of a 300IR daily dose of a sublingual solution of birch pollen allergen extract in adults with allergic rhinoconjunctivitis: results of a double-blind, placebo-controlled study. *Clinical and translational allergy*, *4*(1), 7. https://doi.org/10.1186/2045-7022-4-7
Wu, S., & Xiao, D. (2016). Effect of curcumin on nasal symptoms and airflow in patients with perennial allergic rhinitis. *Annals of allergy, asthma & immunology : official publication of the American College of Allergy, Asthma, & Immunology*, *117*(6), 697–702.e1. https://doi.org/10.1016/j.anai.2016.09.427
Xie, Y., Ju, X., Beaudin, S., Wiltshire, L., Oliveria, J. P., MacLean, J., Sommer, D. D., Cusack, R., Li, O., Banerjee, P., Keith, P. K., O'Byrne, P. M., Bauer, R. N., Staton, T., Gauvreau, G. M., & Sehmi, R. (2021). Effect of intranasal corticosteroid treatment on allergen-induced changes in group 2 innate lymphoid cells in allergic rhinitis with mild asthma. *Allergy*, *76*(9), 2797–2808. https://doi.org/10.1111/all.14835
Xue, C. C., Zhang, A. L., Zhang, C. S., DaCosta, C., Story, D. F., & Thien, F. C. (2015). Acupuncture for seasonal allergic rhinitis: a randomized controlled trial. *Annals of allergy, asthma & immunology : official publication of the American College of Allergy, Asthma, & Immunology*, *115*(4), 317–324.e1. https://doi.org/10.1016/j.anai.2015.05.017
Zangrilli, J., Mansfield, L. E., Uryniak, T., & O'Brien, C. D. (2011). Efficacy of budesonide/formoterol pressurized metered-dose inhaler versus budesonide pressurized metered-dose inhaler alone in Hispanic adults and adolescents with asthma: a randomized, controlled trial. *Annals of allergy, asthma & immunology : official publication of the American College of Allergy, Asthma, & Immunology*, *107*(3), 258–65.e2. https://doi.org/10.1016/j.anai.2011.05.024
Zeiger, R. S., Schatz, M., Li, Q., Solari, P. G., Zazzali, J. L., & Chen, W. (2014). Real-time asthma outreach reduces excessive short-acting β2-agonist use: a randomized study. *The journal of allergy and clinical immunology. In practice*, *2*(4), 445–456.e4565. https://doi.org/10.1016/j.jaip.2014.01.018
Zhu, R., Wang, J., Wu, Y., Yang, Y., Huang, N., Yang, Y., Zhang, R., Ma, D., Yang, L., & Demoly, P. (2019). The Allergic Rhinitis Control Test Questionnaire Is Valuable in Guiding Step-Down Pharmacotherapy Treatment of Allergic Rhinitis. *The journal of*

allergy and clinical immunology. In practice, *7*(1), 272–278. https://doi.org/10.1016/j.jaip.2018.05.028
**General/Internal Medicine**
Bateman, E. D., Reddel, H. K., O'Byrne, P. M., Barnes, P. J., Zhong, N., Keen, C., Jorup, C., Lamarca, R., Siwek-Posluszna, A., & FitzGerald, J. M. (2018). As-Needed Budesonide-Formoterol versus Maintenance Budesonide in Mild Asthma. *The New England journal of medicine*, *378*(20), 1877–1887. https://doi.org/10.1056/NEJMoa1715275
Beasley, R., Holliday, M., Reddel, H. K., Braithwaite, I., Ebmeier, S., Hancox, R. J., Harrison, T., Houghton, C., Oldfield, K., Papi, A., Pavord, I. D., Williams, M., Weatherall, M., & Novel START Study Team (2019). Controlled Trial of Budesonide-Formoterol as Needed for Mild Asthma. *The New England journal of medicine*, *380*(21), 2020–2030. https://doi.org/10.1056/NEJMoa1901963
Bel, E. H., Wenzel, S. E., Thompson, P. J., Prazma, C. M., Keene, O. N., Yancey, S. W., Ortega, H. G., Pavord, I. D., & SIRIUS Investigators (2014). Oral glucocorticoid-sparing effect of mepolizumab in eosinophilic asthma. *The New England journal of medicine*, *371*(13), 1189–1197. https://doi.org/10.1056/NEJMoa1403291
Bieber, T., Simpson, E. L., Silverberg, J. I., Thaçi, D., Paul, C., Pink, A. E., Kataoka, Y., Chu, C. Y., DiBonaventura, M., Rojo, R., Antinew, J., Ionita, I., Sinclair, R., Forman, S., Zdybski, J., Biswas, P., Malhotra, B., Zhang, F., Valdez, H., & JADE COMPARE Investigators (2021). Abrocitinib versus Placebo or Dupilumab for Atopic Dermatitis. *The New England journal of medicine*, *384*(12), 1101–1112. https://doi.org/10.1056/NEJMoa2019380
Brinkhaus, B., Ortiz, M., Witt, C. M., Roll, S., Linde, K., Pfab, F., Niggemann, B., Hummelsberger, J., Treszl, A., Ring, J., Zuberbier, T., Wegscheider, K., & Willich, S. N. (2013). Acupuncture in patients with seasonal allergic rhinitis: a randomized trial. *Annals of internal medicine*, *158*(4), 225–234. https://doi.org/10.7326/0003-4819-158-4-201302190-00002
Cahill, K. N., Katz, H. R., Cui, J., Lai, J., Kazani, S., Crosby-Thompson, A., Garofalo, D., Castro, M., Jarjour, N., DiMango, E., Erzurum, S., Trevor, J. L., Shenoy, K., Chinchilli, V. M., Wechsler, M. E., Laidlaw, T. M., Boyce, J. A., & Israel, E. (2017). KIT Inhibition by Imatinib in Patients with Severe Refractory Asthma. *The New England journal of medicine*, *376*(20), 1911–1920. https://doi.org/10.1056/NEJMoa1613125
Castro, M., Corren, J., Pavord, I. D., Maspero, J., Wenzel, S., Rabe, K. F., Busse, W. W., Ford, L., Sher, L., FitzGerald, J. M., Katelaris, C., Tohda, Y., Zhang, B., Staudinger, H., Pirozzi, G., Amin, N., Ruddy, M., Akinlade, B., Khan, A., Chao, J., … Teper, A. (2018). Dupilumab Efficacy and Safety in Moderate-to-Severe Uncontrolled Asthma. *The New England journal of medicine*, *378*(26), 2486–2496. https://doi.org/10.1056/NEJMoa1804092
Federman, A. D., O'Conor, R., Mindlis, I., Hoy-Rosas, J., Hauser, D., Lurio, J., Shroff, N., Lopez, R., Erblich, J., Wolf, M. S., & Wisnivesky, J. P. (2019). Effect of a Self-management Support Intervention on Asthma Outcomes in Older Adults: The SAMBA Study Randomized Clinical Trial. *JAMA internal medicine*, *179*(8), 1113–1121. https://doi.org/10.1001/jamainternmed.2019.1201
Jackson, D. J., Bacharier, L. B., Mauger, D. T., Boehmer, S., Beigelman, A., Chmiel, J. F., Fitzpatrick, A. M., Gaffin, J. M., Morgan, W. J., Peters, S. P., Phipatanakul, W.,

Sheehan, W. J., Cabana, M. D., Holguin, F., Martinez, F. D., Pongracic, J. A., Baxi, S. N., Benson, M., Blake, K., Covar, R., … National Heart, Lung, and Blood Institute AsthmaNet (2018). Quintupling Inhaled Glucocorticoids to Prevent Childhood Asthma Exacerbations. *The New England journal of medicine*, *378*(10), 891–901. https://doi.org/10.1056/NEJMoa1710988
Johnston, S. L., Szigeti, M., Cross, M., Brightling, C., Chaudhuri, R., Harrison, T., Mansur, A., Robison, L., Sattar, Z., Jackson, D., Mallia, P., Wong, E., Corrigan, C., Higgins, B., Ind, P., Singh, D., Thomson, N. C., Ashby, D., Chauhan, A., & AZALEA Trial Team (2016). Azithromycin for Acute Exacerbations of Asthma : The AZALEA Randomized Clinical Trial. *JAMA internal medicine*, *176*(11), 1630–1637. https://doi.org/10.1001/jamainternmed.2016.5664
Kabashima, K., Matsumura, T., Komazaki, H., Kawashima, M., & Nemolizumab-JP01 Study Group (2020). Trial of Nemolizumab and Topical Agents for Atopic Dermatitis with Pruritus. *The New England journal of medicine*, *383*(2), 141–150. https://doi.org/10.1056/NEJMoa1917006
Krieger, J., Song, L., & Philby, M. (2015). Community health worker home visits for adults with uncontrolled asthma: the HomeBASE Trial randomized clinical trial. *JAMA internal medicine*, *175*(1), 109–117. https://doi.org/10.1001/jamainternmed.2014.6353
Lazarus, S. C., Krishnan, J. A., King, T. S., Lang, J. E., Blake, K. V., Covar, R., Lugogo, N., Wenzel, S., Chinchilli, V. M., Mauger, D. T., Dyer, A. M., Boushey, H. A., Fahy, J. V., Woodruff, P. G., Bacharier, L. B., Cabana, M. D., Cardet, J. C., Castro, M., Chmiel, J., Denlinger, L., … National Heart, Lung, and Blood Institute AsthmaNet (2019). Mometasone or Tiotropium in Mild Asthma with a Low Sputum Eosinophil Level. *The New England journal of medicine*, *380*(21), 2009–2019. https://doi.org/10.1056/NEJMoa1814917
Maurer, M., Rosén, K., Hsieh, H. J., Saini, S., Grattan, C., Gimenéz-Arnau, A., Agarwal, S., Doyle, R., Canvin, J., Kaplan, A., & Casale, T. (2013). Omalizumab for the treatment of chronic idiopathic or spontaneous urticaria. *The New England journal of medicine*, *368*(10), 924–935. https://doi.org/10.1056/NEJMoa1215372
McKeever, T., Mortimer, K., Wilson, A., Walker, S., Brightling, C., Skeggs, A., Pavord, I., Price, D., Duley, L., Thomas, M., Bradshaw, L., Higgins, B., Haydock, R., Mitchell, E., Devereux, G., & Harrison, T. (2018). Quadrupling Inhaled Glucocorticoid Dose to Abort Asthma Exacerbations. *The New England journal of medicine*, *378*(10), 902–910. https://doi.org/10.1056/NEJMoa1714257
Murphy, K., Jacobs, J., Bjermer, L., Fahrenholz, J. M., Shalit, Y., Garin, M., Zangrilli, J., & Castro, M. (2017). Long-term Safety and Efficacy of Reslizumab in Patients with Eosinophilic Asthma. *The journal of allergy and clinical immunology. In practice*, *5*(6), 1572–1581.e3. https://doi.org/10.1016/j.jaip.2017.08.024
Nair, P., Wenzel, S., Rabe, K. F., Bourdin, A., Lugogo, N. L., Kuna, P., Barker, P., Sproule, S., Ponnarambil, S., Goldman, M., & ZONDA Trial Investigators (2017). Oral Glucocorticoid-Sparing Effect of Benralizumab in Severe Asthma. *The New England journal of medicine*, *376*(25), 2448–2458. https://doi.org/10.1056/NEJMoa1703501
O'Byrne, P. M., FitzGerald, J. M., Bateman, E. D., Barnes, P. J., Zhong, N., Keen, C., Jorup, C., Lamarca, R., Ivanov, S., & Reddel, H. K. (2018). Inhaled Combined Budesonide-Formoterol as Needed in Mild Asthma. *The New England journal of medicine*, *378*(20), 1865–1876. https://doi.org/10.1056/NEJMoa1715274

Ortega, H. G., Liu, M. C., Pavord, I. D., Brusselle, G. G., FitzGerald, J. M., Chetta, A., Humbert, M., Katz, L. E., Keene, O. N., Yancey, S. W., Chanez, P., & MENSA Investigators (2014). Mepolizumab treatment in patients with severe eosinophilic asthma. *The New England journal of medicine*, *371*(13), 1198–1207. https://doi.org/10.1056/NEJMoa1403290
Peters, S. P., Bleecker, E. R., Canonica, G. W., Park, Y. B., Ramirez, R., Hollis, S., Fjallbrant, H., Jorup, C., & Martin, U. J. (2016). Serious Asthma Events with Budesonide plus Formoterol vs. Budesonide Alone. *The New England journal of medicine*, *375*(9), 850–860. https://doi.org/10.1056/NEJMoa1511190
Rabe, K. F., Nair, P., Brusselle, G., Maspero, J. F., Castro, M., Sher, L., Zhu, H., Hamilton, J. D., Swanson, B. N., Khan, A., Chao, J., Staudinger, H., Pirozzi, G., Antoni, C., Amin, N., Ruddy, M., Akinlade, B., Graham, N. M. H., Stahl, N., Yancopoulos, G. D., … Teper, A. (2018). Efficacy and Safety of Dupilumab in Glucocorticoid-Dependent Severe Asthma. *The New England journal of medicine*, *378*(26), 2475–2485. https://doi.org/10.1056/NEJMoa1804093
Stempel, D. A., Raphiou, I. H., Kral, K. M., Yeakey, A. M., Emmett, A. H., Prazma, C. M., Buaron, K. S., Pascoe, S. J., & AUSTRI Investigators (2016). Serious Asthma Events with Fluticasone plus Salmeterol versus Fluticasone Alone. *The New England journal of medicine*, *374*(19), 1822–1830. https://doi.org/10.1056/NEJMoa1511049
